# Experiencing weight stigma during childbirth increases the odds of cesarean birth

**DOI:** 10.1186/s12884-025-07251-6

**Published:** 2025-02-21

**Authors:** Regula A. Schwenk, Carmen Wyss, Evelyne M. Aubry

**Affiliations:** 1 Department of Health Professions, Bern University of Applied Sciences, Bern, Switzerland; 2 Department of Health Professions, Bern University of Applied Sciences, Murtenstrasse 10, Bern, 3008 Switzerland

**Keywords:** Delivery, Labor, Pregnancy, Body mass index, Survey, Obesity, Midwifery, Perinatal care, Weight bias

## Abstract

**Background:**

Weight-biased clinical practices and institutional characteristics can have a wide impact on the quality of care provided to women with obesity. This may substantially increase their risks for poor birth outcomes. The current study assessed experienced weight stigma by women during childbirth in maternity care settings in Switzerland. We aimed to identify frequencies, sources, and manifestations of weight-related stigmatization, hypothesizing that such stigma impacts birth outcomes, specifically cesarean birth (CB).

**Methods:**

Data from a nationwide cross-sectional online survey was used to investigate the frequencies, sources, and manifestations of experienced weight stigma during childbirth. Binomial logistic regression was applied to predict CB from experienced weight stigma. Mediation analysis assessed the role of experienced weight stigma in the association between body mass index (BMI) and CB.

**Results:**

In a total of 1352 women who gave birth in the last five years, women with obesity (BMI ≥ 30 kg/m2) experienced weight stigma more often than their peers with healthy weight (BMI 18.5–24.9 kg/m2). Obstetricians were identified as a major source of weight stigma, accounting for 77.8% of stigmatization experienced by women, compared to stigmatization perceived from nurses (21.7%) and midwives (23.8%). Overall, weight stigma was mostly experienced in the form of dismissive or critical comments towards a woman’s figure or weight. Significantly more women with obesity indicated being blamed for weight-related risks during childbirth than their healthy-weighted peers (χ²(2) = 22.2, *P* < 0.001). An increase in the frequency of experienced weight stigma was related to higher odds of intrapartum CB ([aOR], 1.08; 95% CI, 1.02,1.15; *P* < 0.05), and it partially mediated the relationship between increased pre-pregnancy BMI and CB (b = 0.07, SE = 0.029; *P* < 0.05).

**Conclusion:**

Women with obesity reported the highest proportion of weight stigmatization during childbirth, experiencing stigma more frequently than women without obesity. This increased frequency of weight stigma was associated with higher odds of CB. Raising awareness among healthcare providers and reducing potential biases and stigmatization may improve care quality and health outcomes for women with obesity.

## Background

High-quality and respectful care during childbirth is a global priority [1]. However, the actions and interactions of care providers are often susceptible to discriminatory practices against individuals with larger bodies, leading to their marginalization and exclusion [2, 3]. Such weight stigmatization can manifest in various forms, resulting in unequal treatment or inadequate healthcare that may harm the psychological and physical well-being [4]. Studies reveal that over 60% of individuals with larger bodies experience weight stigma originating from healthcare professionals in diverse settings worldwide [5, 6]. In maternity care, increased body mass index (BMI) and excessive gestational weight gain (GWG) have been designated as the strongest predictors of experiencing weight-related stigmatization [7, 8]. With approximately half of all women of reproductive age globally overweight or have obesity, childbirth and the peripartum become a unique period where weight stigma is often experienced [9]. This is particularly evident during regular check-ups, where focus is frequently placed on weight [10].

Studies have emphasized that experiencing weight stigma in maternity care settings has significant implications for psychological and physical health [11, 12]. Even though weight stigmatization experiences do not necessarily have a start and end point and are blended across the entire reproductive life phase, the process of giving birth is considered a key event in the transition to motherhood. Women may be especially vulnerable during this period, as threats to physical and psychological well-being are at their highest [13]. Women during childbirth may have different experiences of weight stigma compared to women during the course of gestation or postpartum. Key features of weight stigmatization by healthcare providers during childbirth include focusing on women’s potentially increased risk of negative birth outcomes related to their weight, denial of respectful or competent treatment, and weight-negative commentary on larger bodies [10, 14]. Experiencing the disrespectful treatment and negative attitudes of a provider can be a barrier to developing a trusting relationship and open communication between woman and healthcare professional, potentially promoting withdrawal and care avoidance [15].

Weight-stigma may further increase women’s physiological stress which can lead to low birth expectations, anxiety, and low self-esteem. This possibly impacts physiological labor processes and increases the risk of adverse birth outcomes [12, 16–18].

To understand the dimensions and negative consequences of weight stigmatization and to improve maternity care accordingly, detailed evidence of the occurrence and implications of weight stigma during childbirth is urgently needed.

Recent research has focused on weight stigma in reproductive health care (e.g., conception and fertility treatment, prenatal and postpartum care), which directly impacts the quality of care and might contribute to higher rates of adverse health outcomes [17–19]. However, a gap remains in studying women experiencing weight stigma during the period of childbirth and its consequences on birth outcomes such as cesarean birth (CB). In a previous study, we have shown that pregnant women with obesity are more likely to experience CB compared to women without obesity [19]. However, much of the association of obesity with CB could not be explained by biomedical mediators, suggesting that the increased odds of CB among women with obesity may be influenced by clinician- and facility-level mediating factors [19]. Providing person-centered care free of weight stigma may improve health outcomes for women with obesity by reducing the number of unnecessary CB. Since healthcare professionals vary in their roles, responsibilities, clinical judgment, and management styles, weight stigma may manifest differently among providers [5, 18]. Identifying how weight stigmatization varies among different healthcare professionals involved in birth care can help tailor interventions to reduce stigma and improve care. In this study, we explored weight stigmatization experienced by women during childbirth. We aimed to identify the frequencies, sources, and manifestations of weight stigmatization, hypothesizing that such stigma is associated with birth outcomes, specifically CB in Swiss maternity care settings.

## Methods

### Study design

We conducted a cross-sectional survey using a self-administered, web-based questionnaire to assess experienced weight stigma during childbirth in Swiss maternity care settings.

### Participants and recruitment

Women living in Switzerland were invited to complete an online survey examining their experienced weight stigma during birth care in clinical settings. Eligible participants were anonymously recruited through online social media platforms, such as Facebook and X using Lime Survey (www.limesurvey.com) for administration. Inclusion criteria were being over 18 years old, having given birth in the last 60 months in a Swiss maternity care unit, being residents of Switzerland, and being able to complete the online questionnaire in German. Data was collected from August to November 2023. Women interested in participating in the anonymous online survey received comprehensive written study information at the beginning of the survey. By clicking the ‘Next’ button to enter the survey, they indicated their willingness to voluntarily take part in this study, providing informed consent for an anonymous online survey. We recognize that not everyone capable of giving birth identifies as female. However, for this study, the term ‘women’ was used to include both women and gender-diverse individuals who give birth.

### Ethical approval

The project was not subject to formal ethical approval as it did not fall under the Swiss Human Research Act (2011), Art. 2, para. 1 [20].

### Data collection

Participants were asked to recall their most recent birth experience focusing on the period from when they entered the obstetric setting for giving birth until their discharge from the care unit. The survey instrument consisted of demographic, medical and obstetric questions that collected the following characteristics: maternal age, education level, gestational age, parity, pregnancy, and birth complications such as gestational diabetes (GDM) (ICD10 024.4) and hypertensive disorders (ICD10 O13, O14, O15.9, O10.9, O11), birth mode including vaginal birth (spontaneous, assisted) or CB (prelabor, performed before the onset of labor or intrapartum, performed during labor), birth care provider, and birth setting. Additionally, participants provided self-reported weight and height data immediately before pregnancy and before birth, which was utilized to compute their pre-pregnancy BMI [21]. Women were considered healthy weight if they had a pre-pregnancy BMI of 18.5–24.9, overweight if they had a BMI of 25–29.9 kg/m2, and obesity was defined as a BMI ≥ 30 kg/m2. For each participant, the GWG (maternal weight at birth minus pre-conceptual weight) was calculated and categorized based on the guidelines of the Institute of Medicine 2009 (IOM) [22]. The IOM recommends a GWG of 11.5 to 16 kg for women of healthy weight, 7 to 11.5 kg for women with overweight, and 5 to 9 kg for women with obesity [22]. Participants exceeding recommendations based on pre-pregnancy BMI were attributed to the group of women exhibiting GWG above IOM guidelines.

In addition, participants responded to three yes or no questions used by Puhl et al. 2021 to assess their experience of weight stigma during childbirth [23]. Those who reported having experienced weight stigma were asked to indicate how frequently and from which healthcare professionals the stigmatization arose. Participants responded to five yes or no items derived from Incollingo Rodriguez et al. 2020 [15]. These items specified the nature of weight stigma experiences (i.e. hearing dismissive or critical comments towards their figure or weight, experiencing intense scrutiny of their weight, being blamed/judged for their weight, having assumptions made about their lifestyle or character, experiencing difficulties fitting medical equipment) in specific reference to their most recent labor. Multiple answers were possible. A group of five women who had given birth within the past five years in Switzerland reviewed the questions. These participants were asked to confirm whether the items were understandable and interpretable as intended. We tested whether participants uniformly understood a question and whether it aligned with the researcher’s intent [24]. Data were securely stored and used exclusively for this research. No third-party access or data sharing occurred.

### Statistical analysis

Descriptive statistics of demographic variables and variables relating to weight stigma were reported.

Inferential statistics included the Cochran’s Q test and pairwise post hoc comparisons to examine whether experienced weight stigma originated differently from various healthcare professionals. Specifically, we assessed the relative contributions of obstetricians, midwives, nurses, and office staff as sources of stigma. Between-BMI group comparisons of the frequency of experienced weight stigma attributed to healthcare professionals were compared by one-way analysis of variance (ANOVA). The Levene test was conducted to test the assumption of homogeneity of variance [25]. In the case of a statistically significant difference between specific groups, Tukey post hoc tests were conducted to analyze which groups differed.

Pearson Chi-Square tests were used to examine the relationship between the manifestation of experienced weight stigma encountered and pre-pregnancy BMI categories. To control for the increased risk of Type I error due to multiple comparisons, we applied a Bonferroni correction to the p-values.

Using binomial logistic regression, odds ratios (ORs) were estimated with 95% confidence intervals (CI)s to assess the association of the frequency of experienced weight stigmatization (FWS), pre-pregnancy BMI, weight gain above IOM recommendations, GDM, and hypertensive disorders with CB (prelabor, intrapartum). The independent variables of interest were either the frequency of experience of weight stigmatization, pre-pregnancy BMI, weight gain above IOM recommendations, gestational diabetes (GDM), or hypertensive disorders. Other women’s characteristics, such as maternal age, education level, parity, and previous cesarean birth history, were included as potential confounders in the model. All analyses were performed in SPSS (Version 28).

Mediation analysis was performed to test whether the FWS serves as a mediator in the relationship between BMI and CB including overall, prelabor, or intrapartum CB. This model allowed us to examine whether the FWS could partially explain how higher pre-pregnancy BMI influences the odds of CB. Using this approach, we tested both the direct and indirect association between BMI and CB, as well as the role of FWS in this relationship. The mediation models were designed using Hayes’ Model 4 in the PROCESS macro with 5000 bootstrapping resamples adopted [26].

## Results

### Sample characteristics

The full sample consisted of 1352 women living in Switzerland who had given birth to their last child no more than 60 months ago. The participants’ mean age was 34.5 (SD, 3.8) years. Approximately 50.1% of women in the sample were primiparous. Around 18.1% of the participants had a BMI > 30 kg/m2, 39.1% gained weight during pregnancy above the IOM recommendations and 23.5% gave birth to their last child by CB.² Regarding education, 1031 (76.3%) of women held a university degree or an equivalent qualification (Table 1).

**Table 1:** Sociodemographic characteristics of 1352 women with or without experienced weight stigmatization

Characteristics	Total	Without experienced WS (*N* = 1209)	With experienced WS (*N* = 143)
**Age**, **mean (SD)**, **y**	34.5 (3.8)	34.6 (3.9)	34.3 (3.6)
**Parity**, **n (%)**			
1	677 (50.1)	601 (49.7)	76 (53.1)
2	504 (37.3)	455 (37.6)	49 (34.3)
3+	171 (12.6)	153 (12.7)	18 (12.6)
**BMI**, **n (%)***(1 missing)*			
< 18.5	44 (3.3)	40 (3.3)	4 (2.8)
18.5–24.9	752 (55.7)	726 (60.1)	26 (18.2)
25-29.9	310 (22.9)	286 (23.7)	24 (16.8)
30≤	245 (18.1)	156 (12.9)	89 (62.2)
**GWG according to IOM recommendations**, **n (%)***(2 missing)*			
Yes	822 (70.9)	756 (62.6)	66 (46.2)
No (above)	528 (39.1)	451 (37.4)	77 (53.8)
**Pregnancy conditions**, **n (%)**			
Gestational diabetes	134 (9.9)	107 (8.9)	27 (18.9)
Hypertensive disorders	104 (7.7)	80 (6.6)	24 (16.8)
**Birth mode**, **n (%)**			
Vaginal *(spontaneous or assisted)*	1034 (76.5)	946 (78.2)	88 (61.5)
CB overall	318 (23.5)	263 (21.8)	55 (38.5)
Prelabor CB	105 (7.8)	91 (7.5)	14 (9.8)
Intrapartum CB	213 (15.8)	172 (14.2)	41 (28.7)
**Education**, **n (%)**			
Lower (primary, secondary)	321 (23.7)	266 (22.0)	55 (38.5)
Higher (tertiary)	1031 (76.3)	943 (78.0)	88 (61.5)

Abbreviations: y, years; n, numbers; WS, weight stigma; CB, cesarean birth; BMI, body mass index; GWG, gestational weight gain; IOM, Institute of Medicine

### Experiences and frequency of weight stigma

In the total sample of women, 10.6% reported having experienced weight stigma during childbirth (143/1352 women). Overall, 36.3% of women with obesity (89/245), 7.7% of women with overweight (24/310), 3.5% of women with a healthy weight (26/752), and 9.1% of women with underweight experienced weight stigma. Of the women who experienced weight stigma during childbirth, 18.2% had a BMI considered healthy, 2.8% were classified as having underweight, 16.8% as having overweight, and 62.2% as having obesity. They were also more likely to have GWG above the IOM recommendations (53.8% vs. 37.4%), experience pregnancy disorders, and have higher rates of CB (38.5% vs. 21.8%) than their non-stigmatized counterparts (Table 1).

The results in Table 2 showed that obstetricians were the most frequently reported source of weight stigmatization for women across all pre-pregnancy BMI categories. Of the women who experienced weight stigma, three-fourths indicated obstetricians as a source of stigmatization (Cochrane’s Q (3) 152.4, *P* < 0.001).

Women who experienced weight stigma reported varying mean FWS during their stay in a birthing unit, with values ranging from 5.6 to 7.3 depending on the healthcare provider involved. The mean FWS were highest in women with obesity. In contrast to the endorsement of the source, the FWS originating from nurses and midwives were higher than from obstetricians (F 25.06; *P* < 0.001). Similar frequency patterns were observed for each pre-pregnancy BMI category in Table 2.

**Table 2:** Sources and frequency of experienced weight (FWS) stigma among the 143 women reporting weight-stigmatizing experiences

	Total (*N* = 143)		Healthy weight (*N* = 26)		Overweight (*N* = 24)		Obesity (*N* = 89)	
Source	Percent endorsed	FWS mean (SD)	Percent endorsed	FWS mean (SD)	Percent endorsed	FWS mean (SD)	Percent endorsed	FWS mean (SD)
Obstetrician	77.80%	5.6 (4.5)	73.10%	5.4 (4.3)	66.70%	4.4 (4.3)	83.10%	6.0 (4.6)
Nurse	21.70%	7.3 (5.1)	15.40%	6.0 (6.1)	20.80%	5.1 (5.7)	24.70%	8.1 (4.8)
Midwife	23.80%	6.7 (5.2)	15.30%	5.8 (6.2)	20.70%	5.2 (5.9)	28.10%	7.2 (5.1)
Office staff	9.10%	7.3 (5.6)	19.20%	6.5 (5.1)	6.70%	3.0 (2.1)	6.70%	9.3 (6.4)
**Statistics**								
P Value	<.001^a^	<.001^b^	<.001^a^	0.01^b^	<.001^a^	0.03^b^	<.001^a^	<.001^b^
Cochran-Q (3)	152.40		29.86		18.00		108.24	
F		25.06		4.62		3.18		18.13

^a^Cochran-Q post-hoc Percentage endorsed (adjusted *P*): (total) obstetrician vs. nurse/midwife/office staff *P* < 0.001, nurse vs. midwife *P* = 0.99, nurse vs. office staff *P* = 0.22, midwife vs. office staff *P* = 0.09; (healthy weight) obstetrician vs. nurse/midwife/office staff *P* < 0.001, nurse vs. midwife *P* = 0.99, nurse vs. office staff *P* = 0.98, midwife vs. office staff *P* = 0.99; (overweight) obstetrician vs. nurse/midwife/office staff *P* < 0.001, nurse vs. midwife *P* = 1.00, nurse vs. office staff *P* = 0.99, midwife vs. office staff *P* = 0.99; (obesity) obstetrician vs. nurse/midwife/office staff *P* < 0.001, nurse vs. midwife *P* = 0.99, nurse vs. office staff *P* = 0.12, midwife vs. office staff *P* = 0.036

^b^Tukey post-hoc frequencies: (total) obstetrician vs. nurse/midwife/office staff *P* < 0.001, nurse vs. midwife *P* = 0.99, nurse vs. office staff *P* = 0.17, midwife vs. office staff *P* = 0.17; (healthy weight) obstetrician vs. nurse/midwife *P* = 0.01, obstetrician vs. office staff *P* = 0.04, nurse vs. midwife *P* = 0.99, nurse vs. office staff *P* = 0.98, midwife vs. office staff *P* = 0.97; (overweight) obstetrician vs. nurse/midwife *P* = 0.17, obstetrician vs. office staff *P* = 0.02, nurse vs. midwife *P* = 0.99, nurse vs. office staff *P* = 0.80, midwife vs. office staff *P* = 0.79; (obesity) obstetrician vs. nurse/midwife/office staff *P* < 0.001, nurse vs. midwife *P* = 0.99, nurse vs. office staff *P* = 0.12, midwife vs. office staff *P* = 0.11

Table 3 provides descriptive information about the manifestation of weight stigmatization experienced across different pre-pregnancy BMI categories. The most common experience of weight stigmatization overall was hearing dismissive or critical comments towards a woman’s figure or weight. Women with obesity more frequently reported that health care providers subjected their weight to intense scrutiny (*X*^*2*^ (2) 13.25, *P* < 0.001). They felt they were being blamed more often for their weight and being faulted for weight-associated risks, which is higher than among their healthy weighted peers (*X*^*2*^ (2) 22.2, *P* < 0.001) (Table 3).

**Table 3:** Descriptive information about manifestations of experienced weight stigma and comparison by BMI categories

Experiences	Total *n* (%)	Healthy weight *n* (%)	Overweight *n* (%)	Obesity *n* (%)	X^2^ (2)	*P* Value
dismissive or critical comments towards a woman’s figure or weight	94 (65.7)	18 (69.2)	16 (66.7)	59 (66.3)	0.098	0.952
intense scrutiny of their weight	56 (39.2)	5 (19.2)^a^	6 (25.0)	45 (50.6)^a^	13.25	< 0.001
Being blamed/judged for weight and related risks	78 (54.5)	4 (15.4)^a^	11 (45.8)	61 (68.5)^a^	22.2	< 0.001
Assumptions made about lifestyle or character	67 (46,9)	13 (50.0)	10 (41.7)	43 (48.3)	0.338	0.845
difficulties fitting medical equipment	22 (15.4)	1 (3.8)	1 (4.2)	20 (22.5)	9.1	0.011

Values within the same row sharing the same letter are significantly different from each other at the Bonferroni-adjusted significance level *P* < 0.0167

### Impact of weight stigma on CB

The results indicated that FWS is a significant predictor of CB (Table 4). Specifically, each additional event of experienced stigma was associated with 7% higher odds of CB ([aOR], 1.07; 95% CI, 1.02,1.14; *P* < 0.05). Intrapartum CB was predicted by the FWS, whereas no significant association was found with prelabor CBs ([aOR], 1.08; 95% CI, 1.02,1.15; *P* < 0.05, and [aOR], 1.03; 95% CI, 0.94,1.12; *P* = 0.57, respectively). Additionally, women with higher pre-pregnancy BMI ([aOR], 1.03; 95% CI, 1.01,1.06; *P* < 0.05), GWG above IOM recommendations ([aOR], 1.44; 95% CI, 1.04,1.98; *P* < 0.05), or hypertensive disorders ([aOR], 2.41; 95% CI, 1.49,3.88; *P* < 0.001) were significantly more likely to give birth by intrapartum CB compared to their peers without these conditions (Table 4).

**Table 4:** Binominal logistic regression models for factors associated with overall, prelabor, and intrapartum CB

Characteristics	CB		Prelabor CB		Intrapartum CB	
	aOR^a^ (95% CI)	*P* Value	aOR^a^ (95% CI)	*P* Value	aOR^a^ (95% CI)	*P* Value
FWS	1.07 (1.02,1.14)	< 0.05	1.03 (0.94,1.12)	0.57	1.08 (1.02,1.15)	< 0.05
Pre-pregnancy BMI	1.04 (1.02,1.07)	< 0.001	1.04 (1.01,1.08)	0.02	1.03 (1.01,1.06)	< 0.05
GWG above IOM recommendations	1.25 (0.95,1.64)	0.10	0.91 (0.59,1.40)	0.66	1.44 (1.04,1.98)	< 0.05
GDM	0.81 (0.51,1.26)	0.51	0.69 (0.33,1.46)	0.33	0.92 (0.55,1.53)	0.74
Hypertensive disorders	1.93 (1.24,3.01)	< 0.001	0.81 (0.36,1.85)	0.62	2.41 (1.49,3.88)	< 0.001

Abbreviations: FWS, frequency of experienced weight stigma; BMI, body mass index; IOM, Institute of Medicine (REF); GWG, gestational weight gain; GDM, gestational diabetes mellitus; CB, cesarean birth; aOR, adjusted Odds Ratio; CI, confidence interval

^a^adjusted for all variables in the table, maternal age, education levels, parity, previous CB

The mediation model showed both direct and indirect positive associations between pre-pregnancy BMI and CB (Table 5). Specifically, the FWS significantly partially mediated the relationship between increased pre-pregnancy BMI and CB. Higher pre-pregnancy BMI was associated with more FWS (b = 0.131; SE = 0.009; *P* < 0.001), and the FWS was related to an increased likelihood of CB (b = 0.065; SE = 0.028; *P* < 0.05). A significant positive association was also observed between FWS and intrapartum CB (b = 0.07, SE = 0.029; *P* < 0.05). However, no significant association was found between FWS and prelabor CB (b = 0.013, SE = 0.043; *P* = 0.29). This result indicated partial mediation, with FWS significantly contributing to the association between BMI and CB but suggesting that additional factors may also influence this relationship.

**Table 5:** Mediation model of the effect of pre-pregnancy BMI on frequency of experienced weight stigma, and CB

Effects	b (effect)	SE (Bootstrapping)	CI 95% (Bootstrapping)	*P* Value
BMI direct effect on FWS	0.131	0.009	(0.112, 0.151)	< 0.001
BMI direct effect on CB (total)	0.047	0.012	(0.024, 0.070)	< 0.001
FWS direct effect on CB (total)	0.065	0.028	(0.011,0.119)	< 0.05
BMI indirect effect on CB (total) through FWS	0.009	0.004	(0.001, 0.161)	
BMI direct effect on prelabor CB	0.036	0.017	(0.027, 0.096)	< 0.05
FWS direct effect on prelabor CB	0.013	0.043	(-0.072,0.096)	0.29
BMI indirect effect on prelabor CB through FWS	0.002	0.006	(-0.012,0.019)	
BMI direct effect on intrapartum CB	0.042	0.013	(0.014, 0.068)	< 0.001
FWS direct effect on intrapartum CB	0.07	0.029	(0.014,0.127)	< 0.05
BMI indirect effect on intrapartum CB through FWS	0.009	0.004	(0.001,0.017)	

Abbreviations: BMI, body mass index; FWS, frequency of experienced weight stigma; CB, cesarean birth; SE, standard error; CI, confidence interval

## Discussion

### Experienced weight stigma

While the occurrence of weight stigma during pregnancy and postpartum has been subject to several studies, we provide first evidence of weight stigma that was experienced specifically during the period of childbirth [11, 15, 27]. In this sample, one in ten women reported that they had experienced weight stigma in Swiss birth care settings. This seems to be less frequent than in the study of Incollingo Rodriguez et al. where they reported experienced weight stigma in almost 20% of women during pregnancy and postpartum in the United States [15]. As women with obesity most commonly experience weight stigma, the lower proportion of women experiencing weight stigma in our study may be explained by the relatively low prevalence of obesity among women of childbearing age in Switzerland [27–29].

The period around childbirth is also shorter than the entire perinatal phase. This shorter duration could reduce the opportunities for encountering weight stigmatization. Childbirth might be a time when women prioritize safety and having technically proficient healthcare professionals over experiencing sensitive interpersonal treatment [27]. Moreover, Puhl and Brownell have highlighted that the memory of weight-related discrimination can diminish over time [30]. Women participating in this study may not have accurately recalled or may have underreported experiences of weight stigma, especially if they perceived these encounters as less impactful at the time.

Our results further indicate that women with obesity affirmed having experienced weight stigmatization during childbirth more often than those in other BMI groups. In our sample, 36.3% of women with obesity reported feeling judged, shamed and blamed for their weight during birth, which is consistent with what is reported throughout perinatal care services in the United States [15]. However, the consequences of experiencing weight stigma during childbirth are concerning, as women’s experience of birth care is critical for achieving satisfaction and meeting positive expectations [18]. This is important for avoiding long-term negative effects on maternal health [31]. Experiencing or anticipating negative attitudes, discrimination, and weight bias from healthcare professionals during labor may impact women’s self-esteem and empowerment, affecting their ability to cope with labor [32, 33]. In addition, weight-biased care can cause high levels of stress, which in turn increases maternal anxiety and pain perception, affecting labor progress and birth outcomes [34, 35].

### Source and manifestations of weight stigma

Women across all BMI groups identified obstetricians as the primary source of experienced weight stigma in Switzerland. Compared to nurses or midwives, obstetricians were reported as a source of weight stigmatization about three and a half times more frequently. Additionally, our analysis revealed that women experienced weight stigma primarily in the form of dismissive or critical comments towards their figure or weight. Recent reviews have offered recommendations for reducing weight stigmatization in health care such as the attendance of courses on obesity and weight-related bias in addition to basic training [36]. In Sweden, although similar proportions of obstetricians and midwives reported receiving education on obesity, midwives were one-third more likely to have been trained in improving their communication skills for weight sensitivity [5]. In several studies, healthcare providers expressed their difficulties in communicating about weight [37–39]. Healthcare providers are aware that talking about body weight may be a sensitive topic for women with obesity and bringing up the subject should be based on the women’s best interests regarding their needs [5]. Lack of confidence, insufficient communication skills, and weight-biased beliefs may result in stigmatizing comments that while well-intended might not be conducive to maintaining a woman’s self-esteem [15]. In an Australian sample, 60% of the birth care providers reported that confidence in providing advice to childbearing women with obesity was the main barrier to providing good quality care [37]. Education and training in supporting women with obesity were regarded as inadequate or non-existent [37]. Offering training modules designed to enhance effective and sensitive communication about body weight may be crucial to improve optimal care for pregnant women with obesity and potentially for all women struggling with weight gain during pregnancy.

The results of our study further suggest that, compared to other women, women with obesity more frequently encounter conversations where the focus is on weight and weight-related health risks. Since women with obesity are considered at high risk for complicated births, receiving information regarding risks remains common during perinatal care [40]. However, risk-focused consultations are often perceived as stigmatizing actions by women with obesity [32]. They reported feeling blamed for their weight, guilty about their obesity, and worried about its impact on their baby’s health [41]. On the one hand, women with obesity find it important to be informed about the health risks and consequences associated with higher weight [42, 43]. On the other hand, they may experience anxiety about complication risks due to higher weight [44, 45]. Women emphasized that they want to be informed about risk factors, but they do not want this information to result in being automatically classified as at risk or diagnosed with a complication that has not yet occurred [46]. Risk-focused care can cause stress for women with obesity [47]. Clinical guidelines for high-risk management of obesity in birth care may even increase the likelihood of weight stigma by emphasizing women’s body size [27]. Actions that follow standardized care guidelines that focus solely on promoting risk reduction and safety may categorize and violate the integrity of women with obesity [43]. Our results also suggest that significant societal barriers, including negative misperceptions about personal responsibility for higher weight, are perceived as highly stigmatizing by women with obesity. A review of UK newsprint media found that 73% of the media used pejorative language when discussing obesity, often blaming women for health risks to themselves, their children, and the healthcare system [48]. Such media narratives, often encountered by healthcare professionals, perpetuate and normalize weight stigma [9]. Additionally, women with obesity in this study indicated experiencing weight stigma due to structural barriers, such as inadequate access to resources. Similarly, a previous qualitative study found that examination tables were often too narrow, gowns were too small, and waiting room furniture was perceived as fragile and unaccommodating [49].

A lack of inclusive care environments, with properly sized medical equipment, and sufficient time for sensitive discussions may hinder healthcare professionals from providing stigma-free birth care.

### Weight stigma is associated with CB

Although obesity is not a medical indication for performing a CB, women with obesity are more likely to experience CB compared to women without obesity [19, 50]. Even in women without any medical risk factors, obesity seems to be associated with increased rates of CB [19, 51]. While pathways through biomedical intervening factors such as comorbidities, or large-for-gestational-age infants appear to contribute to an increased CB rate in women with obesity, they only explain little of the association of obesity with CB [19]. We extended this knowledge by demonstrating a positive association between BMI and CB, mediated by the frequency of experienced weight stigma. Despite evidence that obesity significantly impacts the mode of childbirth, there has been limited research on whether and to what extent the intrapartum care environment could be improved to minimize poor birth outcomes [52]. The caregiving environment during labor includes multiple decisions, communication patterns, and clinical judgments that may vary by type of provider and management style. Professional weight-biased opinions of obesity as a pathological condition may lead to the stigmatization of women, resulting in suboptimal decision-making and inadequate care [46]. A cohort study of nearly 12’000 childbirths showed that the way women with obesity were managed in labor had a significant biasing effect on the relationship between pre-pregnancy BMI and CB rates [53]. The conditions under which labor and childbirth occur, such as high time pressure, complexity, and cost containment may even increase the likelihood that the resulting care will be poorly matched to the needs of women with higher weight [54]. Further research is needed to determine the appropriate labor management and optimal care for women with obesity during childbirth.

A predominantly risk-focused approach to professional support, combined with biased communication about weight-related risks during childbirth and structural barriers such as inadequate access to resources can directly compromise the quality of care. These factors represent potential pathways through which weight stigma could contribute to higher rates of adverse birth outcomes [55].

### Strengths and limitations

Strengths and limitations of this study need to be acknowledged. Recruiting participants through social media enabled us to reach a large sample of women. Although randomized sampling is typically required for broad generalization, social media recruitment has several benefits compared to traditional approaches. It includes faster recruitment, better-targeted participant selection, and strong privacy measures [56, 57]. However, the study’s generalizability may be restricted due to potential sampling bias. Our sample consists primarily of highly educated, German-speaking women who live in Switzerland, and are social media users. The higher proportion of women with higher education levels in this study may have influenced the representation of experienced weight stigma. Women with lower education levels often belong to lower socioeconomic strata, which is associated with increased odds of maternal obesity and a higher likelihood of experiencing weight stigma [58]. However, the prevalence of maternal obesity in our study was higher and the CB rate lower than in the general female population of childbearing age in Switzerland [19, 29]. This may have confounded the influence of weight stigma on CB.

In addition, certain groups are more vulnerable to the harmful consequences of weight stigma. For example, information on the ethnic/racial diversity of the women in this study was not available. Yet, racial and ethnic norms may also drive weight stigma differently and influence its perception [59]. To achieve a generalizable and comprehensive understanding, future research evaluating the role of weight stigma in labor management and on birth outcomes will require more diverse samples. Knowledge about experienced weight stigma across the general birthing population will be crucial in healthcare settings to provide quality care for all women.

A further limitation of our study is the reliance on self-reported weight data. While participants were encouraged to reference maternity documents or other records for accuracy, not all participants may have had access to their records. In cases where documentation was unavailable, participants were advised to estimate their weight, which may have affected the precision of the analyzed data. Although we emphasized the importance of accurate reporting and assured participants of anonymity, the variability in weight recall methods remained a potential source of imprecision. Future studies could mitigate this limitation by incorporating verified clinical weight measurements when feasible.

Finally, recall bias may have occurred, as women’s ability to recall experiences of weight stigma during childbirth over five years may have been limited, potentially influencing the results of this study. More research is needed to further explore the perceptions of weight stigma, which may evolve over time.

## Conclusion

This study highlights the weight stigma experienced by women during childbirth and its associations with CB in maternity care settings in Switzerland, which pose challenges to the provision of quality care. Particularly in the context of labor and childbirth, experiencing weight stigma appears to be common for women with obesity. Weight-biased attitudes and behaviors among maternity care providers may contribute to the adoption of risk-focused care management and overmedicalized birthing practices when caring for this group of women [60]. Weight stigma from healthcare professionals may affect women’s birth outcomes by increasing the odds of CB. Ultimately, this influences downstream health outcomes in the postpartum period and later pregnancies [46]. In preventing poor birth outcomes in women with obesity, attention should be paid not only to the physiological effects of obesity but also to psychosocial contributing factors, such as frequently experienced weight stigmatization in maternity care settings. As the significant consequences of weight stigma in health care become clearer, effective strategies to reduce discriminatory behavior based on weight should be implemented.

Our findings suggest that there is a need for providing continuing education to care providers focusing on the complexity and consequences of weight stigma to improve effective sensitive communication during birth care [61].

To prevent weight stigma, it may be beneficial to consider the multiple and complex contexts of care challenges for women with obesity when developing maternity care policies and guidelines, with the goal of women-centered practice and needs-based birth care [62, 63] Weight stigma can be pronounced in birth care settings, with significant consequences for maternal health and well-being. Addressing this issue by promoting a stigma-free birth environment is critical, as it lays the foundation for respectful, safe, and supportive maternity care and improves birth outcomes for women and their children.

## Data Availability

The datasets used and/or analysed during the current study are available from the corresponding author on reasonable request.

## Abbreviations

BMI: Body mass index
CI: Confidence intervals
CB: Cesarean birth
FWS: Frequency of experienced weight stigma
GDM: Gestational diabetes
GWG: Gestational weight gain
IOM: Institute of Medicine
n: Numbers
SD: Standard deviation
